# First description of the male of *Volesusnigripennis* Champion, 1899, with new records from Ecuador and Panama, taxonomical notes, and an updated key to the genera of Sphaeridopinae (Hemiptera, Reduviidae)

**DOI:** 10.3897/zookeys.841.31153

**Published:** 2019-05-02

**Authors:** Hélcio R. Gil-Santana, Jader Oliveira

**Affiliations:** 1 Laboratório de Diptera, Instituto Oswaldo Cruz, Av. Brasil, 4365, 21040-360, Rio de Janeiro, RJ, Brazil Laboratório de Diptera, Instituto Oswaldo Cruz Rio de Janeiro Brazil; 2 Laboratório de Parasitologia, Universidade Estadual Paulista “Julio de Mesquita Filho”, Faculdade de Ciências Farmacêuticas UNESP/FCFAR, Rodovia Araraquara Jaú, KM 1, 14801- 902, Araraquara, SP, Brazil Universidade Estadual Paulista “Julio de Mesquita Filho” Araraquara Brazil

**Keywords:** Heteroptera, male genitalia, Neotropics, Salyavatinae, *
Sphaeridops
*, *
Veseris
*

## Abstract

The genus *Volesus* Champion, 1899 is redescribed and the male of *V.nigripennis* Champion, 1899 is described for the first time and found to be similar to the female in both structure and coloration. The genus and the species are recorded from Ecuador and Panama for the first time. Notes on the taxonomic history of Sphaeridopinae and an updated key to the genera are provided.

## Introduction

Recent papers have documented new records of reduviid genera and species for several Neotropical countries (e.g. Forero 2006, Gil-Santana 2007, 2008, Melo 2008, Inés and Coscarón 2009, Dellapé et al. 2010).

Froeschner (1981, 1999) provided catalogs of Heteroptera, including Reduviidae, recorded from Ecuador and Panama, respectively. Further records of Reduviidae from Ecuador and Panama were provided by Maldonado (1990) and in papers describing or reviewing different taxa of this family (e.g. Dougherty 1995, Martin-Park et al. 2012, Zhang et al. 2016).

The cladistic analysis of Weirauch (2008) showed that Salyavatinae and Sphaeridopinae are a monophyletic group, while studies by Weirauch (2008) and Gordon and Weirauch (2016) provided evidence that Salyavatinae is paraphyletic and Sphaeridopinae is a sister group to the genus *Salyavata* Amyot & Serville, 1843 (Salyavatinae). Here we are considering Salyavatinae and Sphaeridopinae as separate subfamilies (following e.g. Weirauch et al. 2014, Gil-Santana et al. 2015).

Sphaeridopinae includes *Sphaeridops* Amyot & Serville, 1843 with three species, *Veseris* Stål, 1865 with two species and *Volesus* Champion, 1899 monotypic with *V.nigripennis* Champion, 1899 (Gil-Santana et al. 2000, Gil-Santana and Alencar 2001, Forero 2004, Gil-Santana et al. 2015).

A summary of the scant data on biology of Sphaeridopinae was provided by Gil-Santana et al. (2015).

In the present paper, notes on the taxonomical history of Sphaeridopinae are provided, clarifying some inconsistencies regarding nomenclature and taxonomical changes. *Volesus* is redescribed and the male of *V.nigripennis* is described for the first time. The genus and the species are recorded from Ecuador and Panama for the first time. Based on the results obtained here, an updated key to the genera of Sphaeridopinae is presented.

## Material and methods

**Figures 1–3.**
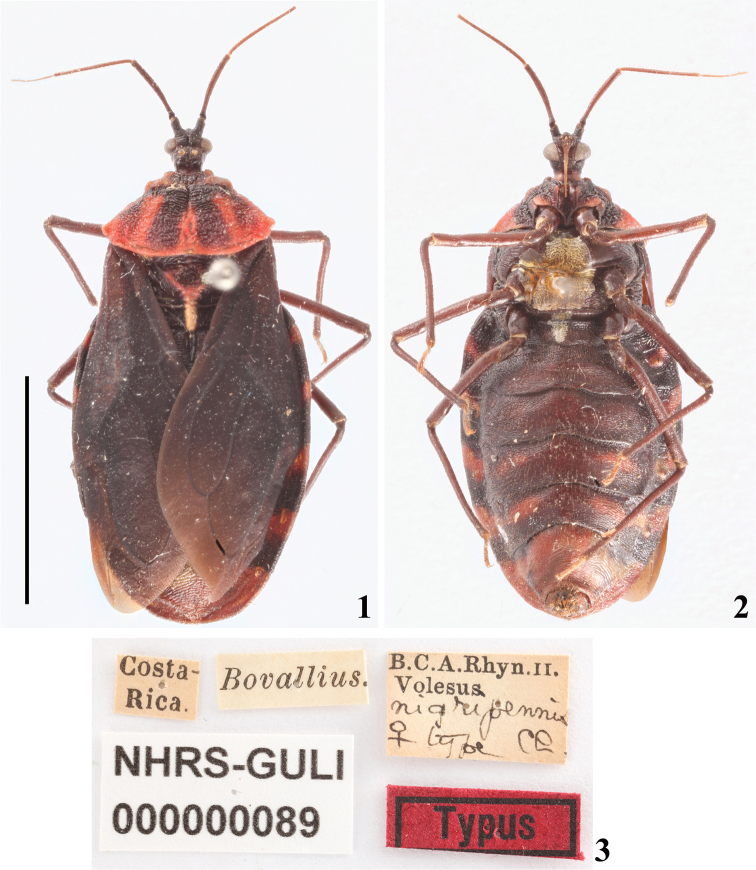
*Volesusnigripennis*, female, holotype deposited in NRM, catalog number NHRS-GULI000000089, photographed by Gunvi Lindberg, © 2018 Naturhistoriska riksmuseet. Made available by the Swedish Museum of Natural History under Creative Commons Attribution 4.0 International Public License, CC-BY 4.0, https://creativecommons.org/licenses/by/4.0/legalcode. **1** dorsal view **2** ventral view **3** labels. Scale bar: 10 mm (**1**).

Photographs of the holotype of *Volesusnigripennis* Champion, 1899 (Figs 1–3), which is deposited at the Swedish Museum of Natural History (NRM), Stockholm, Sweden, were kindly provided by Dr Gunvi Lindberg (NRM).

Data on a female of *V.nigripennis* from Panama and deposited in the National Museum of Natural History (NMNH), Smithsonian Institution, Washington, DC, USA, were kindly provided by Dr Silvia A. Justi (The Walter Reed Biosystematics Unit, WRBU, Smithsonian Institution, Museum Support Center), with the support of Dr Thomas Henry and James N. Zahniser (NMNH).

Scanning electron microscopy images (Figs 7–8, 12–13, 15, 17–19, 21–23, 29–34, 36–40, 46) were obtained by the second author (JO). A male of *V.nigripennis* and its external genitalia were cleaned in an ultrasound machine. Subsequently, the samples were dehydrated in alcohol, dried in an incubator at 45 ºC for 20 min, and fixed in small aluminum cylinders with transparent glaze. Sputtering metallization was then performed on the samples for 2 minutes at 10 mA in an Edwards sputter coater. After this process, the samples were studied and photographed using a high-resolution field emission gun scanning electron microscope (FEG-SEM; JEOL, JSM-7500F), as described by Rosa et al. (2010, 2014).

All remaining figures were produced by the first author (HRG-S). The fixed adults, microscopic preparations and genitalia were photographed using digital cameras (Nikon D5200 with a Nikon Macro Lens 105 mm, Sony DSC-W830). Drawings were made using a camera lucida. Images were edited using Adobe Photoshop CS6.

Observations were made using a stereoscope microscope (Zeiss Stemi) and a compound microscope (Leica CME). Measurements were made using a micrometer eyepiece. The total length of the head was measured excluding the neck, for better uniformity of this measurement. Dissections of the male genitalia were made by first removing the pygophore from the abdomen with a pair of forceps and then clearing it in 20% NaOH solution for 24 hours. Following this procedure, the phallus was recorded without inflation (Figs 48–50). The endosoma was then everted (Figs 51, 52) by carefully pulling on the endosoma wall, using a pair of fine forceps. The dissected structures were studied and photographed in glycerol.

General morphological terminology mainly follows Schuh and Slater (1995). The terminology of the genitalia structures follows Lent and Wygodzinsky (1979). However, the “vesica”, as recognized by the latter authors, has been considered as absent in reduviids. The assumed equivalent structure in reduviids is a somewhat sclerotized appendage of endosoma (Forero and Weirauch 2012) but not the homologous vesica of other heteropterans, such as Pentatomomorpha (Rédei and Tsai 2011). Thus, this term is not used here. Yet, we adopted the denomination of paired membranous lobes on the endosoma, lateral to the dorsal phallothecal sclerite, from Weirauch (2008), to the flat paired expansions of the endosoma wall (Fig. 50). On the other hand, in order to maintain uniformity with the general terminology followed here, the basal plate bridge is named as such and not as ponticulus basilaris as in Weirauch (2008).

The specimens described here will be deposited in the Entomological Collection of the Oswaldo Cruz Institute (“Coleção Entomológica do Instituto Oswaldo Cruz”), Rio de Janeiro (CEIOC) and in the Dr Jose Maria Soares Barata Triatominae Collection (CTJMSB) of the São Paulo State University Julio de Mesquita Filho, School of Pharmaceutical Sciences, Araraquara, São Paulo, Brazil.

When citing the text on the labels of a pinned specimen, a slash (/) separates the lines and a double slash (//) different labels. All measurements are in millimeters (mm).

## Results

### Taxonomy

#### Subfamily Sphaeridopinae Amyot & Serville, 1843

Amyot and Serville (1843) created the “Groupe” “Sphéridopides” in the “tribu” “Brevicipites” to include only *Sphaeridops* described by them to accommodate the species *Reduviusamoenus* Lepeletier & Serville, 1825. Walker (1873a) recorded Sphaeridopidae with only *Sphaeridops* included in it. Interestingly, in the same volume this genus is further keyed out with *Veseris* Stål, 1865 as belonging to the subfamily “Acanthaspida”. The characteristics mentioned by Walker (1873a) to separate these genera were the same mentioned by Stål (1865, 1872). Walker (1873b) recognized the family “Sphaeridopidae, Serv.”, clearly referring to page 381 in which it was established by Amyot and Serville (1843).

*Sphaeridops* was regarded as belonging to Acanthaspidinae (e.g. Stål 1872, Lethierry and Severin 1896), in which *Veseris* and *Volesus* Champion, 1899 were also included when described (Stål 1865, Champion 1899).

Pinto (1927) established Sphaeridopidae as a new family, containing *Sphaeridops* and *Limaia* Pinto, 1927, described in the same paper. Interestingly, Pinto (1927) argued that he was adopting the opinion of Amyot and Serville (1843) that *Sphaeridops* should be part of a separate family sensu “Brevicipites”, without mentioning the similarity between the etymology of Sphaeridopidae and “Sphéridopides”, neither the references of Walker (1873a, b) to it. Pinto (1927) also claimed that the name “Brevicipites” could not prevail according to nomenclatural rules, because it was not based on a genus name, and instead included it as a synonym of the new family Sphaeridopidae. The group has been subsequently considered as a subfamily and most authors credited its authorship to Pinto (1927) (e.g. Costa Lima 1940, Wygodzinsky 1949, Maldonado 1990, Forero 2004), but Putshkov and Putshkov (1985) attributed the authorship of Sphaeridopinae to Amyot and Serville (1843) (referring to “Sphaeridopides”). Costa Lima (1940) in a general book on Brazilian Heteroptera, stated the synonym between *Limaiaruber* Pinto, 1927 under *Veserisrugosicollis* (Stål, 1862), without giving any reasons for the proposed synonym. In order to review this synonymy, a search for the male type of *L.ruber* in the Entomological Collection of Oswaldo Cruz Institute, Rio de Janeiro, where it should be deposited (Pinto 1927) was performed (Gil-Santana et al. 1999), but it was not located. Nevertheless, although Maldonado (1990) had credited to Wygodzinsky (1949) the above-mentioned synonymy, it was undoubtedly firstly stated by Costa Lima (1940).

On the other hand, the synonym between *Limaia* and *Veseris* Stål, 1865 was in fact first recorded by Wygodzinsky (1949).

Similarly, Costa Lima (1940) was the first to record *Sphaeridopspallescens* (Walker, 1873) (described as *Reduviuspallescens*) as a junior synonym of *S.amoenus* and not Wygodzinsky (1949) as recorded by Maldonado (1990).

It is noteworthy that *Eurylochusbellator* Torre-Bueno, 1914 and *Volesusnigripennis* Champion, 1899 were first mentioned as belonging to Sphaeridopinae by Costa Lima (1940). *Eurylochus* Torre-Bueno, 1914 was considered a junior synonym of *Veseris* by Gil-Santana and Alencar (2001).

Therefore, Sphaeridopinae currently includes three exclusively Neotropical genera: *Sphaeridops*, *Veseris* and *Volesus* (Gil-Santana et al. 2015).

Pinto (1927) provided the following diagnosis for Sphaeridopidae: a short head, without an anteocular portion; large antenniferous tubercles, clearly exceeding the anterior border of the head; eyes large, salient, almost touching each other on the ventral portion of the head; and the labium straight, with three [visible] segments.

Maldonado and Santiago-Blay (1992) considered that Sphaeridopinae are characterized by two unique characters: the head mostly occupied by the very large eyes, and the antennifers raised on the vertex, close together, between the eyes. These authors were the first to argue that the Sphaeridopinae have a few other unusual characters: presence of sensory organs on the fore lobe of the pronotum (unknown function) and the fact that the dorsal and ventral components of connexivum are well separated by a vertical sclerite; these characteristics were recorded in *Sphaeridopseulus* Maldonado & Santiago-Blay, 1992. Maldonado and Santiago-Blay (1992) assumed that the smooth areas on the fore lobe of pronotum were sensory organs derived from SEM images of them. These authors also commented that they had observed “corresponding organs” in other two genera, without stating which ones. Gil-Santana et al. (1999) and Gil-Santana and Alencar (2001) recorded sensory organs on fore lobe of pronotum in both species currently included in *Veseris*. However, these latter authors based their conclusions only on the macroscopic aspect of similar smooth structures of fore lobe, without using SEM imaging.

Schuh and Slater (1995) diagnosed Sphaeridopinae by the following set of characters: head projecting only slightly beyond the anterior margin of eyes; eyes large, nearly contiguous ventrally; antennae inserted on anteriorly projecting tubercles; rostrum straight; all tarsi three-segmented. Weirauch et al. (2014) considered that Sphaeridopinae are characterized by a large, robust body; large eyes almost covering the entire head; and a short, straight, thin labium. The keys to the genera provided by Gil-Santana and Alencar (2001), Forero (2004) and Gil-Santana et al. (2015) included a different set of characteristics.

##### Volesus

Taxon classificationAnimaliaHemipteraReduviidae

Champion, 1899

Volesus Champion, 1899: 296 [description, comments on systematic relationship with other genera]; Wygodzinsky 1949: 65 [catalog]; Putshkov and Putshkov 1985: 99 [catalog]; Maldonado 1990: 490 [catalog]; Schuh and Slater 1995: 158 [citation]; Gil-Santana et al. 1999: 2 [citation]; Gil-Santana and Alencar 2001: 96, 100 [citation, key]; Forero 2004: 164 [diagnosis], 189 [key]; Forero 2006: 36 [citation]; Weirauch et al. 2014: 101 [citation]; Gil-Santana et al. 2015: 336 [citation], 337 [key].

###### Type species.

*Volesusnigripennis* Champion, 1899, by monotypy.

###### Diagnosis.

*Volesus* can be separated from other genera of Sphaeridopinae by the combination of characters presented in the key below, and additionally by the following characteristics: eyes medium-sized, not covering the head; interocular distance larger than the width of eye, dorsally, and approximately equivalent to it, ventrally; labium with only two visible segments.

###### Redescription.

Body integument shiny, generally diffusely rugose, with linear irregular impressions more intensively and coarsely in thorax, except on lateral portions of mesosternum and median portions of some sternites, in which it is mostly smooth. *Head* subrectangular in dorsal view, moderately elongate in lateral view; transverse sulcus straight, moderately impressed meeting eyes at inner posterior angle; a midlongitudinal well-marked sulcus running from transverse sulcus to approximately level of anterior margin of eyes; antenniferous stout, cylindrical, diverging forward, straight apically; anteocular region curved downwards, not, or barely, visible in dorsal view; eyes medium-sized, interocular distance in dorsal view larger than width of an eye; labium with only two visible segments; first visible labial segment short, enlarged; second visible segment long, thin, straight. *Thorax*: pronotum trapezoidal; fore lobe much shorter and narrower than hind lobe of pronotum; transverse (interlobar) sulcus indistinct; median longitudinal sulcus ill defined, short, running on approximately basal fourth of hind lobe and separated from the median transverse depression of fore lobe by an irregular, curved carina. Prosternum somewhat depressed, with a pair of acute short, lateral processes, directed forward, median portion mostly occupied by stridulitrum, shortly prolonged posteriorly on midline, not surpassing the level of posterior margin of fore coxae and continuous with adjacent sclerite; meso- and metasternum flattened; fore coxae close, separate by a distance smaller than width of each coxa; middle and hind coxae separated from each other by a distance approximately equivalent to slightly more than twice width of each of them. Femora, tibia and tarsi slender, segments with similar width in all three pairs of legs; femora with a small ventral subapical protuberance; a small spongy fossa on apices of fore and mid tibiae. Tarsi three segmented. *Abdomen* enlarged at about middle portion; small scars of dorsal abdominal glands openings (dag) on medial anterior margins of tergites IV–VI; a vertical sclerite separating dorsal and ventral components of connexivum. Sternites with canaliculae (carinulate) on anterior margin of some segments.

###### Distribution.

Colombia, Costa Rica, Ecuador (**new record**), Panama (**new record**).

##### Volesus nigripennis

Taxon classificationAnimaliaHemipteraReduviidae

Champion, 1899

Figs 1–3 , 4–8 , 9–13 , 14–19 , 20–25 , 26–28 , 29–34 , 35–39 , 40–44 , 45–48 , 49–52 , 53–57

Volesus nigripennis Champion, 1899: 296 [description], Tab. XVIII [Figure 14]; Costa Lima 1940: 207 [citation], Wygodzinsky 1949: 65 [catalog]; Maldonado 1990: 490 [catalog]; Gil-Santana et al. 1999: 2 [citation]; Forero 2004: 164 [citation from Colombia], Figures 5.25, 5.103; Forero 2006: 36 [new record from Colombia], Figures 56–57; Gil-Santana et al. 2015: 336 [citation].

###### Notes.

*Volesusnigripennis* was described based on a female from Costa Rica (Champion 1899). The female holotype is deposited at the Swedish Museum of Natural History (NRM), Stockholm, Sweden, and its photos are available on their website (Figs 1–3).

Forero (2004, 2006) recorded this species from Colombia, based on a unique female. These two females have been the only specimens of *V.nigripennis* known so far. Forero (2004) argued that the knowledge of the male of the species would be useful to a definition in relation to other members of Sphaeridopinae.

Additionally, a female specimen of *V.nigripennis* from Panama was located in the collection of the NMNH. Upon our request, Dr Silvia A. Justi (WRBU) examined the specimen, sent us photos of it and provided the data on the labels, which are transcribed below. The specimen was identified by the Late P. Wygodzinsky. Although it had been previously coated with metal for electronic microscopy, the identification of the specimen is still possible and represents a new record of this species for Panama.

###### Material examined.

*Volesusnigripennis*. **ECUADOR, Esmeraldas**, Tundaloma Lodge, near Calderón, 01.18277N, 078.75259W (01°10'57"N 78°45'09"W), 55m a.s.l., 8–9.ii.2014, A. Kury & A. Giupponi *leg.*, 1 male (CEIOC), 1 male (CTJMSB).

###### Additional specimen.

*Volesusnigripennis*. **PANAMA**: Escobal Road / Atl. Canal Zone / 24 VI [19]74 [handwritten] / Col: D. Engleman // Drake Colln. ex / J. Maldonado C. / Coll 1996 [characters partially cut off at the bottom of the label] // *Volesus* [handwritten] / *nigripennis* [handwritten] / Champion [handwritten] / Wygodzinsky [det.], 1 female (NMNH).

###### Description.

**Figures 4–8.**
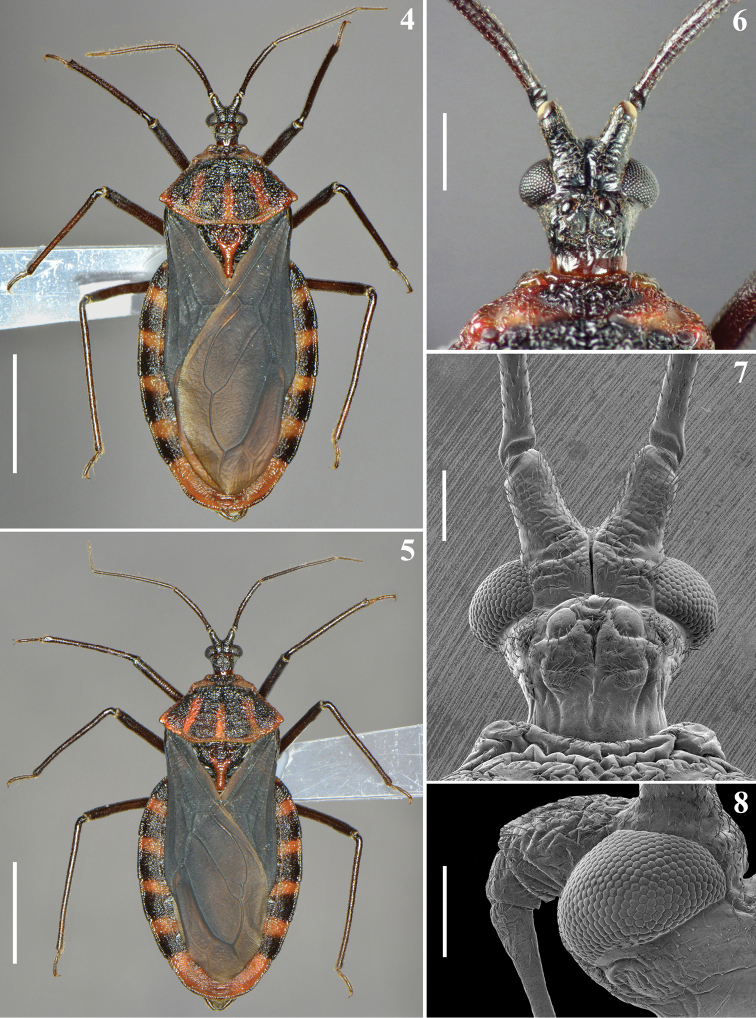
*Volesusnigripennis*, male **4–5** dorsal view **6–8** head **6–7** dorsal view **8** except distal half of second visible labial segment, lateral view. Scale bars: 5.0 mm (**4–5**); 1.0 mm (**6**); 0.5 mm (**7–8**).

**Figures 9–13.**
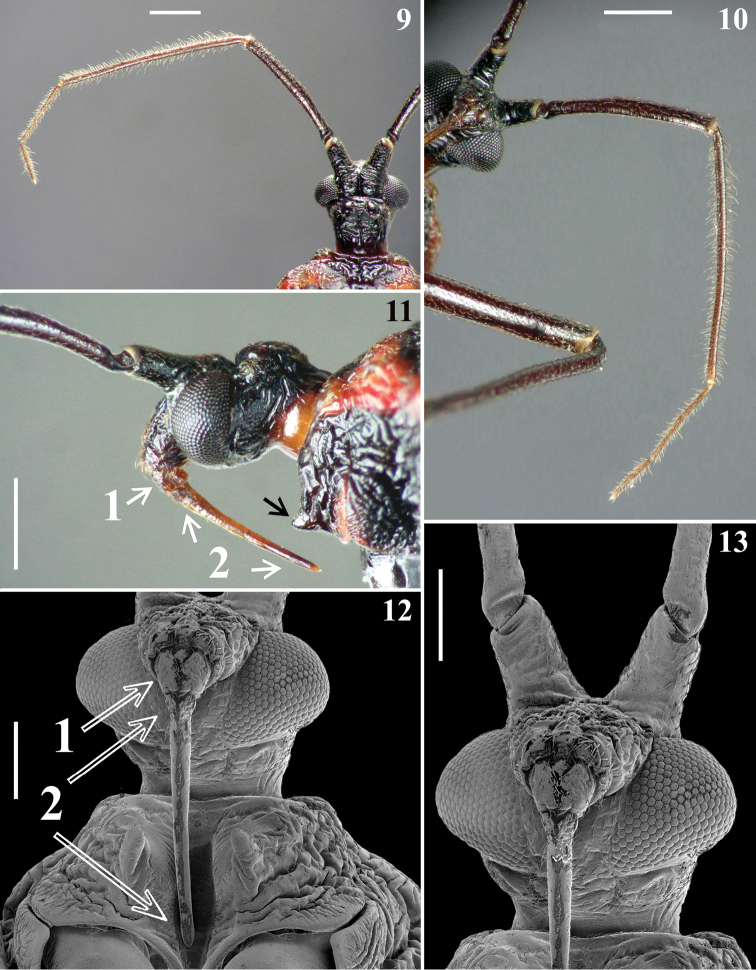
*Volesusnigripennis*, male **9** head and left antenna, dorsal view **10** left antenna, ventral view **11** head and fore lobe of pronotum, lateral view (black arrow points to prosternal process; first and second visible labial segments indicated by white arrows) **12, 13** ventral view **12** head, except antenniferous, and most part of prosternum (arrows point to first and second visible labial segments) **13** head, except distal half of second visible labial segment. (**1** first **2** second visible labial segments). Scale bars: 1.0 mm (**9–11**); 0.5 mm (**12, 13**).

**Figures 14–19.**
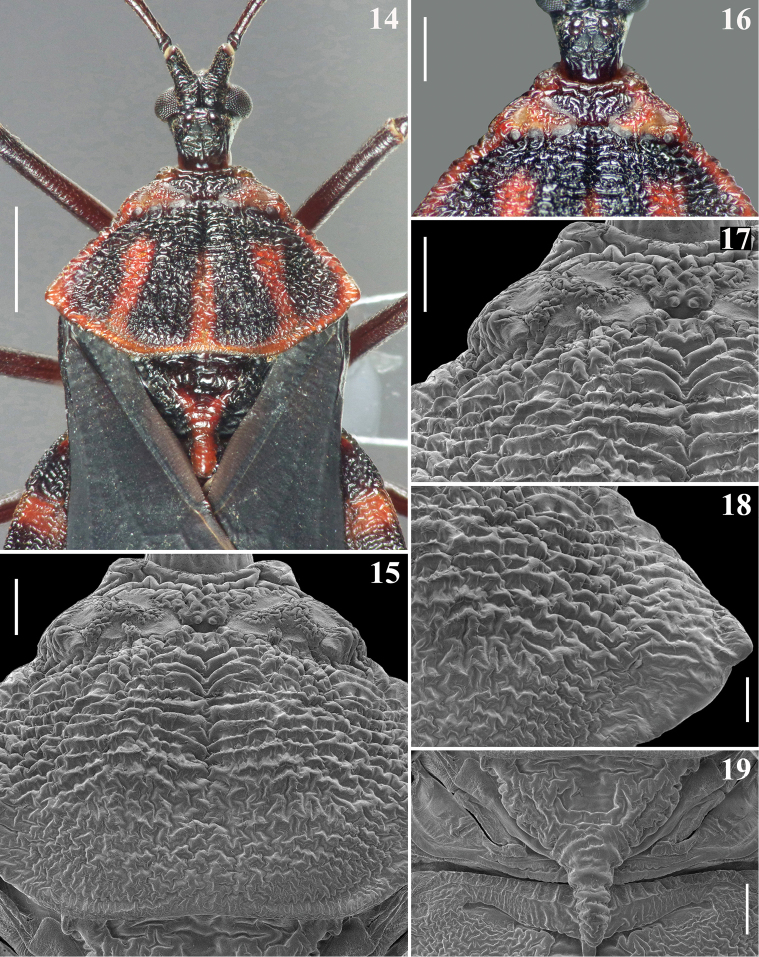
*Volesusnigripennis*, male, dorsal view **14** head, pronotum, scutellum and basal portions of hemelytra and connexivum **15–18** pronotum **15** median portion **16–17** fore lobe and basal portion of hind lobe **17** left side and midline **18** lateral right portion, including humeral angle **19** scutellum. Scale bars: 2.0 mm (**14**); 0.5 mm (**15–17, 19**); 0.3 mm (**18**).

**Figures 20–25.**
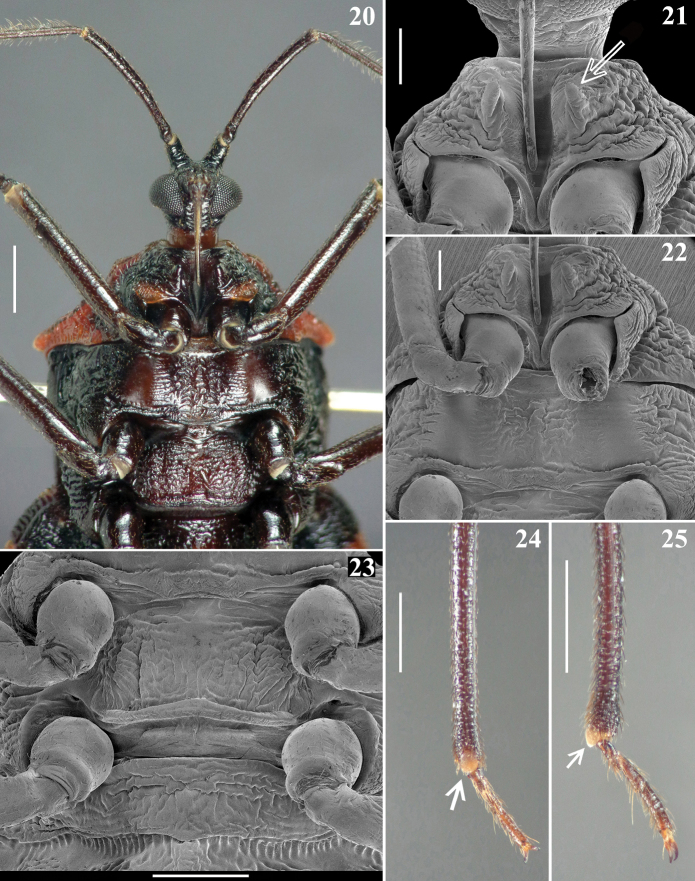
*Volesusnigripennis*, male **20–23** ventral view **20** head and thorax **21** prothorax, arrow points to prosternal process **22** prothorax and mesosternum **23** metasternum, middle and hind coxae, and median portion of base of abdomen **24, 25** apices of tibiae, arrow points to spongy fossa **24** fore tibia, ventral view **24** middle tibia, lateral view. Scale bars: 1.0 mm (**20, 23–25**) 0.5 mm (**21, 22**).

***Male.*** (Figs 4–57). Measurements: total length to tip of abdomen: 16.9–17.3; to tip of forewings: 16.1–16.5; head (excluding neck, measured in lateral view) length: 2.2; length of anteocular portion (measured in lateral view): 0.5; length of postocular portion (measured in lateral view): 0.7; width across eyes: 1.8; interocular distance, dorsal view: 0.9, ventral view: 0.5–0.6; width of eye, dorsal view: 0.5; ventral view: 0.6; length of eye: 0.6–0.7; distance between external margin of ocelli: 0.7–0.8; distance between ocelli: 0.25; maximum width of ocellus: 0.2–0.25; length of antennifer: 0.7; lengths of antennal segments: I: 2.5; II: 3.8; III: 1.5; IV: 0.9; lengths of labial segments, first visible: 0.3; second visible: 1.7–1.8. Thorax: pronotum: fore lobe, length: 0.8; maximum width: 3.2; hind lobe: length 3.0; maximum width: 5.9; scutellum, length: 2.3; width: 2.7; length of process: 1.1–1.2; length of hemelytra: 12.5. Fore legs: length of femur: 3.8; length of tibia: 4.8–4.7; length of spongy fossa: 0.25; length of tarsus: 1.2–1.3; middle legs, length of femur: 4.5–4.6; length of tibia: 4.8–5.1; length of spongy fossa: 0.25; length of tarsus: 1.2–1.3; hind legs: length of femur: 5.2–5.3; length of tibia: 6.3–6.7; length of tarsus: 1.3. Abdomen, length: 12.5; maximum width: 7.7–7.8.

**Figures 26–28.**
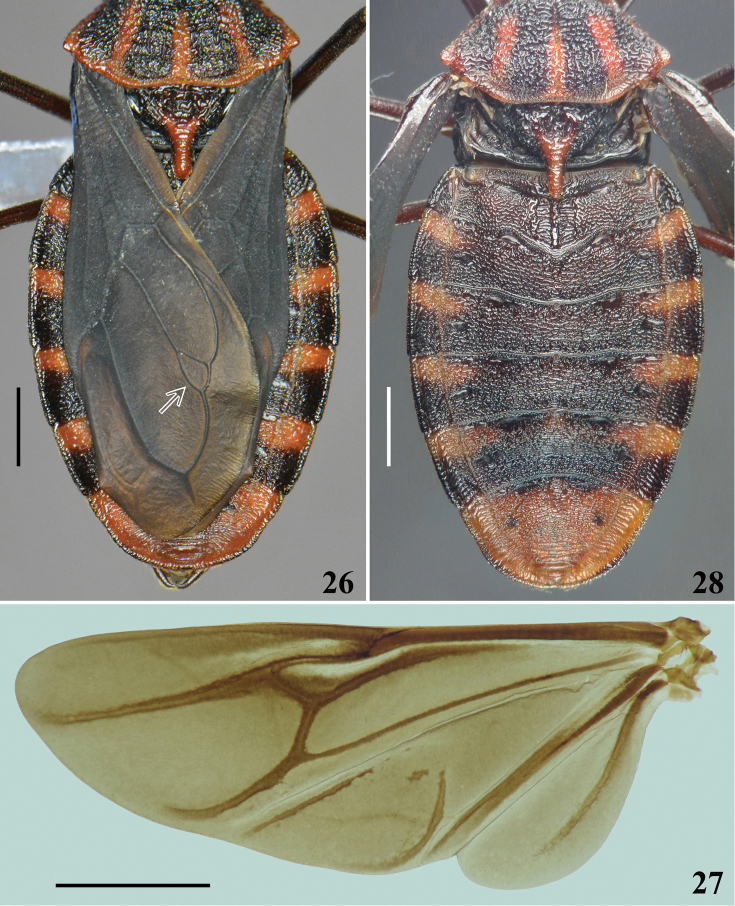
*Volesusnigripennis*, male, dorsal view **26** distal portion of pronotum, scutellum, hemelytra and connexivum, arrow points to a small additional cell at approximately apical fourth of the cubital vein **27** left hind wing **28** distal portion of thorax and abdomen (wings moved away). Scale bar: 2.0 mm.

*Coloration*: general coloration blackish with reddish markings (Figs 4–5, 14, 20, 26, 28, 35). Head generally blackish; neck mostly reddish; apices of antenniferous tubercles pale; antennal segment II brownish black; antennal segments III–IV brownish; labium brownish (Figs 4–6, 9–11, 14, 20). Thorax blackish, brownish black on meso- and metasternum, with the following reddish thoracic markings: on anterior collar and their projections; on lateral and posterior margins of pronotum; on most of fore lobe of pronotum, except its median portion; on hind lobe of pronotum, a median and a pair of lateral converging bands, which are continuous with reddish posterior margin, ending approximately at mid and anterior thirds of hind lobe, respectively; and on postero-superior portion (approximately) of propleura and process of scutellum (Figs 4–6, 14, 16, 20, 26, 28). Legs generally blackish; spongy fossa on fore and mid tibiae somewhat paler (Figs 4–5, 20, 24–25). Hemelytra black, somewhat paler, brownish, on approximately distal half of clavus, medially and about distal half of the membrane, except veins and area just surrounding them (Figs 4–5, 26). Hind wing generally brownish, with veins darkened (Fig. 27). Abdomen blackish to blackish brown; tergite VI with a median reddish spot just below anterior margin; tergite VII almost completely reddish, blackish on and just below anterior margin and with a pair of rounded blackish spots on mid-lateral portion (Fig. 28). Connexivum reddish on: extreme base of segment II, approximately basal third of segments III–V, and somewhat less than basal half of segment VI; connexival portion of segment VII almost entirely reddish with only posterior border of approximately distal half darkened; ventrally, marking on segment II is a small spot on external margin; on segments III–VI connexival reddish markings are prolonged dorsally to a short distance on lateral portion of respective tergite as a subtriangular marking, and ventrally, as a somewhat curved lateral marking, directed backwards, reaching spiracles, which are surrounded by reddish posterior margin; sternite II with anterior margin and median portion, on approximately distal half reddish to reddish brown; transverse median bands, on sternites III–VII, progressively larger, reddish brown in one specimen and pale brownish in other, joining lateral reddish markings described above in sternites V–VII, the latter almost completely reddish, with dark coloration restricted to anterior margin and adjacent to genital capsule (Figs 4–5, 26, 28, 35). Exposed portion of pygophore and parameres blackish (Fig. 35).

*Vestiture*: body generally covered by sparse short, somewhat curved, adpressed, thin, golden to brownish setae. Head: eyes, ocelli and neck glabrous; region adjacent to insertion of labium with more numerous and somewhat longer setae; ventral surface of first visible labial segment and basal portion of second visible labial segment moderately setose, dorsal surface of correspondent portions with fewer setae; additionally, some sparse setae scattered on the proximal third of second visible segment, remainder glabrous. Antenna: segment I sparsely covered with setae similar to those of general vestiture but slightly longer, more numerous at apex; segments II–IV densely setose, covered with scattered longer, somewhat curved, brownish setae and much more numerous shorter, thinner, whitish setae (Figs 9, 10). Thorax. Some longer straight thin setae on posterior margin of pronotum adjacent to lateral portion of scutellar base; setae are sparser on ventral surface; smooth lateral areas of mesosternum glabrous. Hemelytra: small adpressed setae sparsely scattered on corium, more numerous at its apex; apical two thirds of clavus, respective adjacent area of corium and membrane glabrous. Legs generally with similar vestiture of the body; setae longer and thicker on tibiae, becoming more numerous towards apex; tarsi with stiff, pale, yellowish to golden-yellowish, oblique to curved setae, with variable lengths. Abdomen: tergites I–V almost completely glabrous, with some scattered small darkened or pale setae, almost imperceptible; tergite VI with some more numerous pale setae; tergite VII with scattered longer golden setae. Connexivum: lateral margins with numerous adpressed short curved darkened setae, forming a few irregular rows; these setae become somewhat longer and paler on distal margin of segment VII; segments II–VI dorsally glabrous; some sparse setae on dorsal surface of distal third of segment VII. Sternites generally covered with sparse thin golden to pale setae; somewhat longer and more numerous setae on median portion of segments VI–VII and on pygophore, except its middle portion.

**Figures 29–34.**
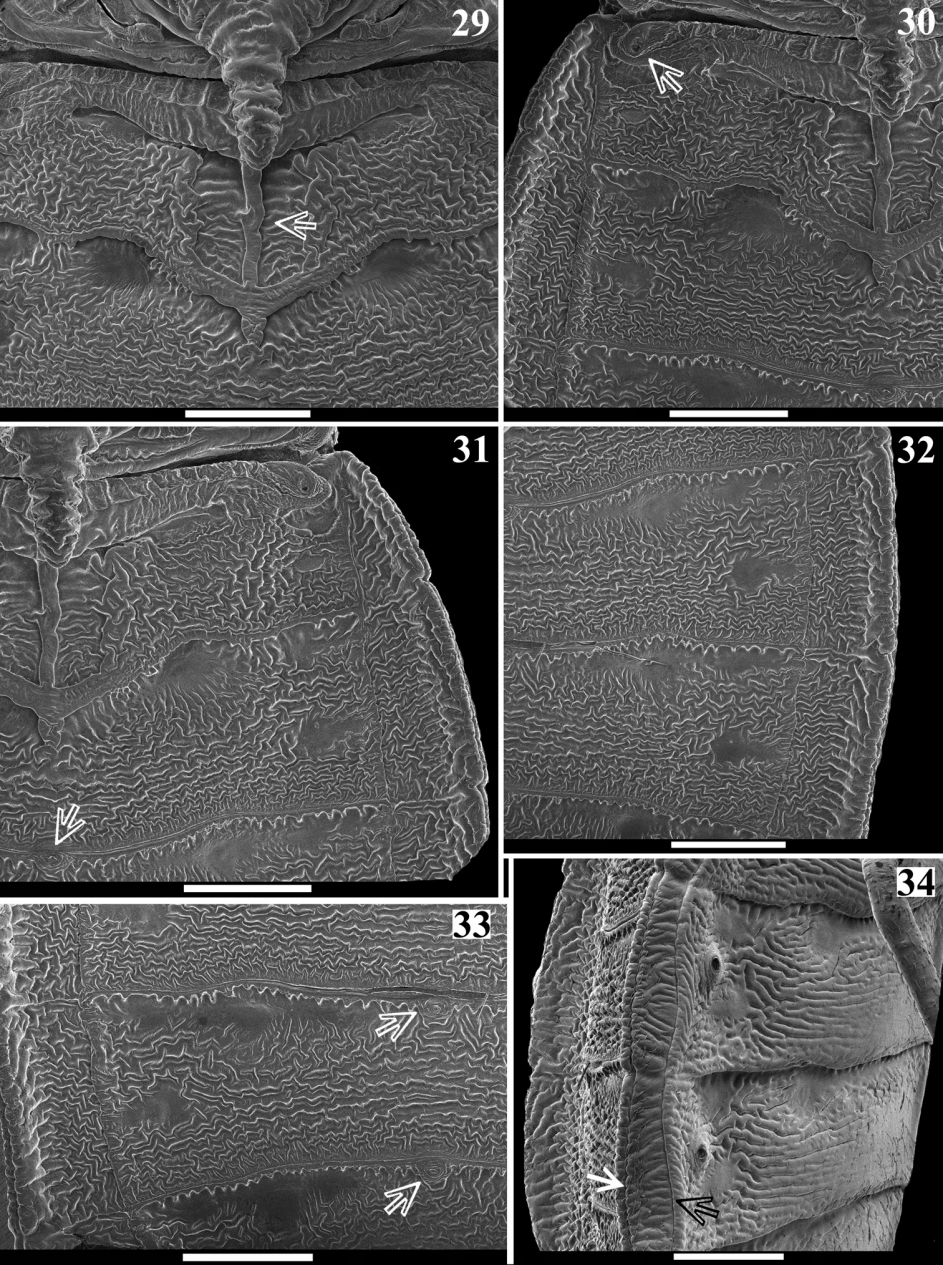
*Volesusnigripennis*, male **29–33** dorsal view **29** process of scutellum, median portions of tergites I–II and basal half of tergite III, arrow points to midlongitudinal keel of tergite II **30–31** mediolateral portions of tergites I–III **30** arrow points to first (dorsal) abdominal spiracle **31** arrow points to the **dag** on tergite IV **32** lateral portions of tergites III (distal part), IV–V **33** mediolateral portions of distal part of tergite IV, tergite V and basal part of tergite VI, arrows point to the **dag** on tergites V and VI **34** abdominal segments III-IV, lateral view, arrows point to the vertical sclerite of connexivum. (**dag** scar of dorsal abdominal gland opening). Scale bar: 1.0 mm.

**Figures 35–39.**
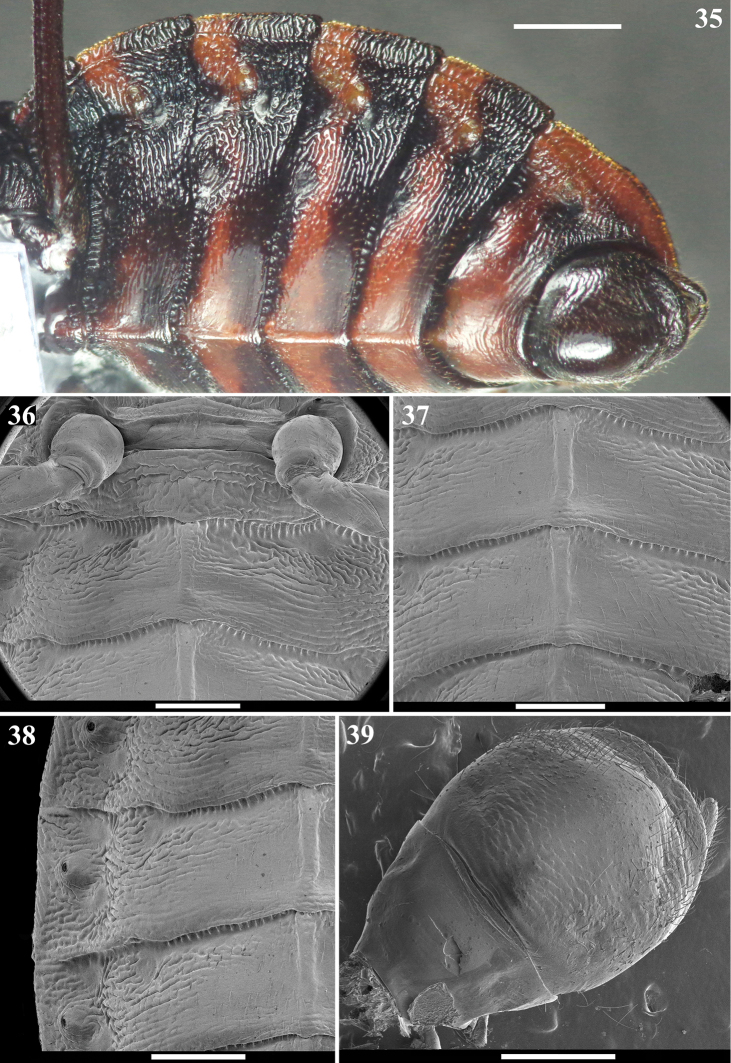
*Volesusnigripennis*, male **35** abdomen, lateroventral view **36–39** ventral view **36** hind coxa, median portions of sternites II–III and basal portion of sternite IV **37** median portion of distal margin of sternite III, sternites IV–V and anterior margin of sternite VI **38** mediolateral portions of distal half of sternite III and sternites IV–V, except lateroposterior angle of the latter **39** segment VIII and genital capsule detached from abdomen. Scale bars: 2.0 mm (**35**); 1.0 mm (**36–39**).

**Figures 40–44.**
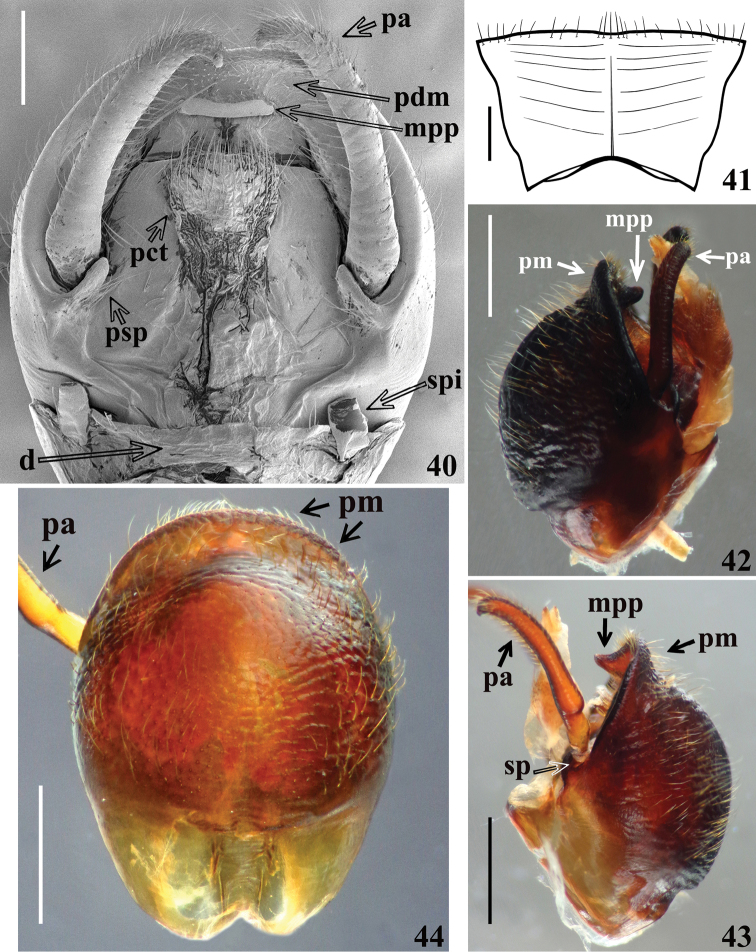
*Volesusnigripennis*, male **40** distal half of segment VIII and genital capsule, dorsal view (**d** dorsal portion of segment VIII; **pct** proctiger; **pdm** posterior dorsal margin of pygophore; **psp** medial prolongation of the socket of the insertion of the paramere; **spi** spiracle of segment VIII) **41** segment VIII, ventral view **42, 43** genital capsule, lateral view **44** pygophore, ventral view. (**mpp** median process of pygophore; **pa** paramere; **pm** posterior margin of pygophore; **sp** socket of the insertion of the paramere. Scale bars: 0.5 mm (**40, 41**); 1.0 mm (**42–44**).

*Structure*: Head. Anteocular portion slightly shorter than postocular portion (in lateral view); ocelli separated by a distance slightly larger than transverse width of each ocellus, positioned medially to level of inner posterior angle of eyes and close to transverse sulcus; antenniferous large; first antennal segment slightly longer than head, stout, somewhat curved, its approximately basal fourth slightly thinner; remaining antennal segments progressively thinner, cylindrical; labium reaching or surpassing the mid third of stridulitrum (Figs 6–14, 20–22). Thorax. Anterior collar inconspicuous; anterolateral angles rounded and small (Figs 15, 16); fore lobe with irregular areas with smooth and whitish integument; a median transverse depression on fore lobe present between medial margins of longer curved smooth areas (Figs 14–17); humeral angles acute, slightly prominent (Figs 14, 18); posterior margin of hind lobe slightly curved on middle third (Figs 14, 15). Scutellum sculptured, median depression shallow, process stout, horizontal, apex rounded (Figs 14, 19). Distance between acute prosternal processes: 0.7. Hemelytra generally dull; on extreme base of dorsal surface, laterally, and on lateral portion, basally, moderately shiny; not reaching tip of abdomen, ending somewhat apically to level of the mid third of seventh tergite (Figs 4–5, 26); in one specimen, the membrane has a small additional cell at approximately apical fourth of cubital vein (Fig. 26). Abdomen. Integument generally also rugose (Figs 28–34), except on median portions of sternites IV–VII, in which it is mostly smooth (Figs 34–38). Connexivum largely exposed, laterally to hemelytra (Figs 4–5); anterior margin of tergite I carinulate (Figs 29–31); tergite II with a mid-longitudinal keel and median third of posterior margin curved backwards (Figs 28–31). Sternites carinulate on anterior margin of segments III–V in one specimen and also on segment VI in the other; on sternite III, canaliculae are somewhat larger and extend more towards lateral portion, occupying approximately two thirds of anterior margin, except midline; on following segments canaliculae become progressively slightly smaller and occupy approximately median third of anterior margin, except midline; a median shallow keel on distal two thirds of segment II and somewhat more elevated in sternites III–VI (Figs 35–38). Segment VIII not visible externally, sclerotized on ventral portion, which becomes somewhat wider towards posterior margin; latter almost straight and with a few short setae; dorsal portion membranous and narrower; spiracles on dorsal margin of ventral portion (Figs 39–41).

**Figures 45–48.**
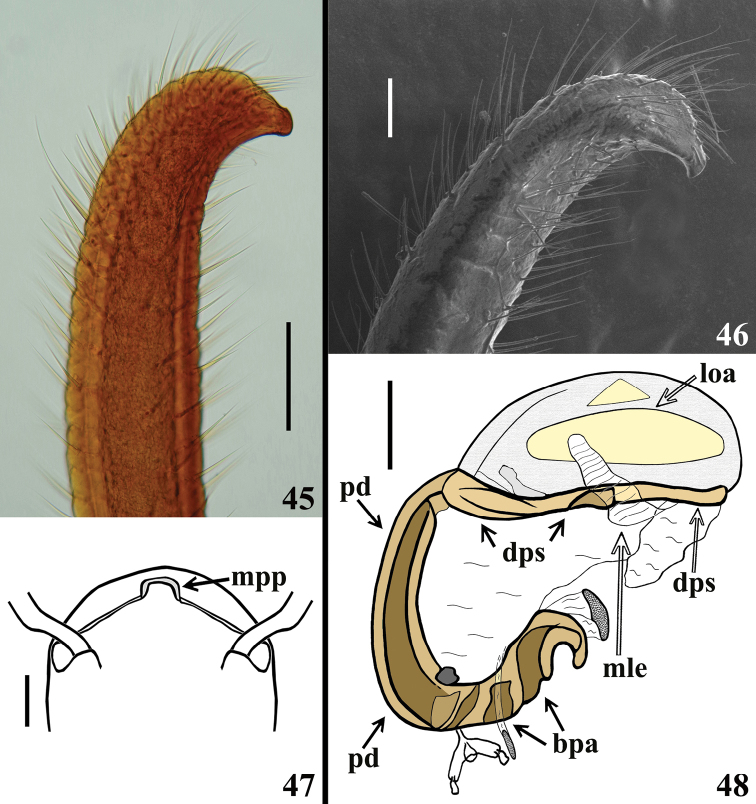
*Volesusnigripennis*, male genitalia **45, 46** apical portion of paramere, lateral view **47** apical portion of pygophore, anterior view (setae omitted). (**mpp** median process of pygophore) **48** phallus not inflated, lateral view. (**bpa** basal plate arm; **dps** dorsal phallothecal sclerite; **loa** lateral oval area; **mle** membranous lobe on endosoma; **pd** pedicel). Scale bars: 0.2 mm (**45**); 0.1 mm (**46**); 0.5 mm (**47, 48**).

**Figures 49–52.**
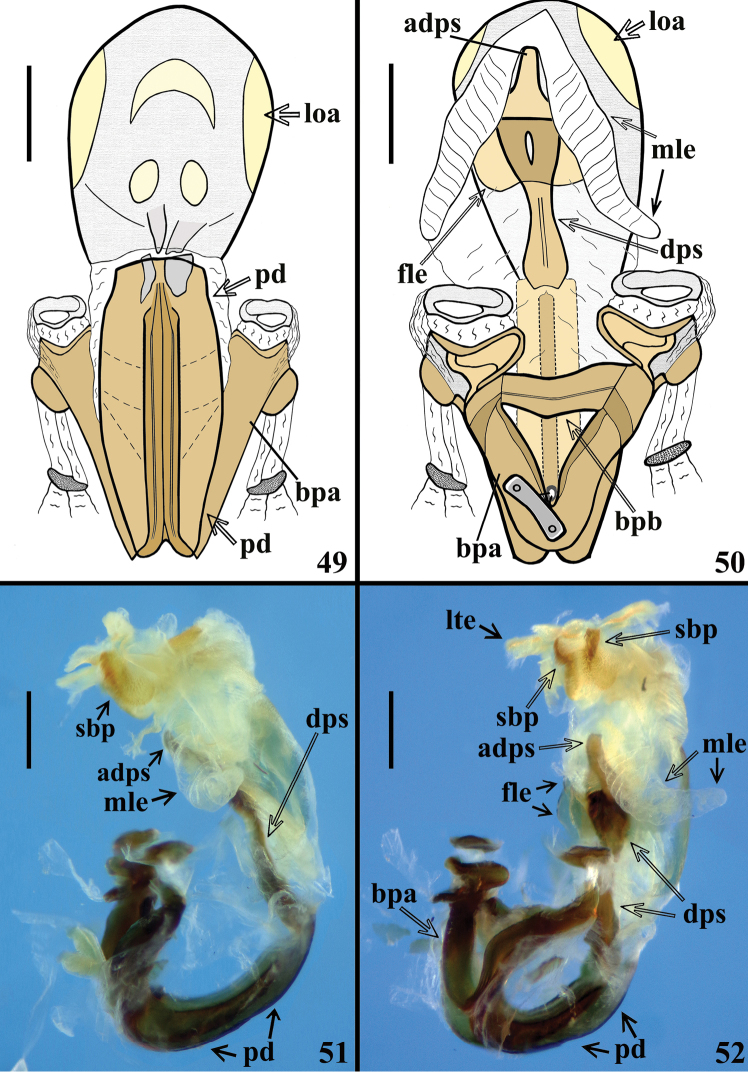
*Volesusnigripennis*, male genitalia, phallus **49, 50** not inflated **49** ventral view **50** dorsal view **51, 52** inflated **51** lateral view **52** laterodorsal view. (**adps** apex of dorsal phallothecal sclerite; **bpa** basal plate arm; **bpb** basal plate bridge; **dps** dorsal phallothecal sclerite; **fle** flat lateral expansion; **loa** lateral oval area; **lte** lateral tubular short expansion; **mle** membranous lobe on endosoma; **pd** pedicel; **sbp** subapical process). Scale bar: 0.5 mm.

**Figures 53–57.**
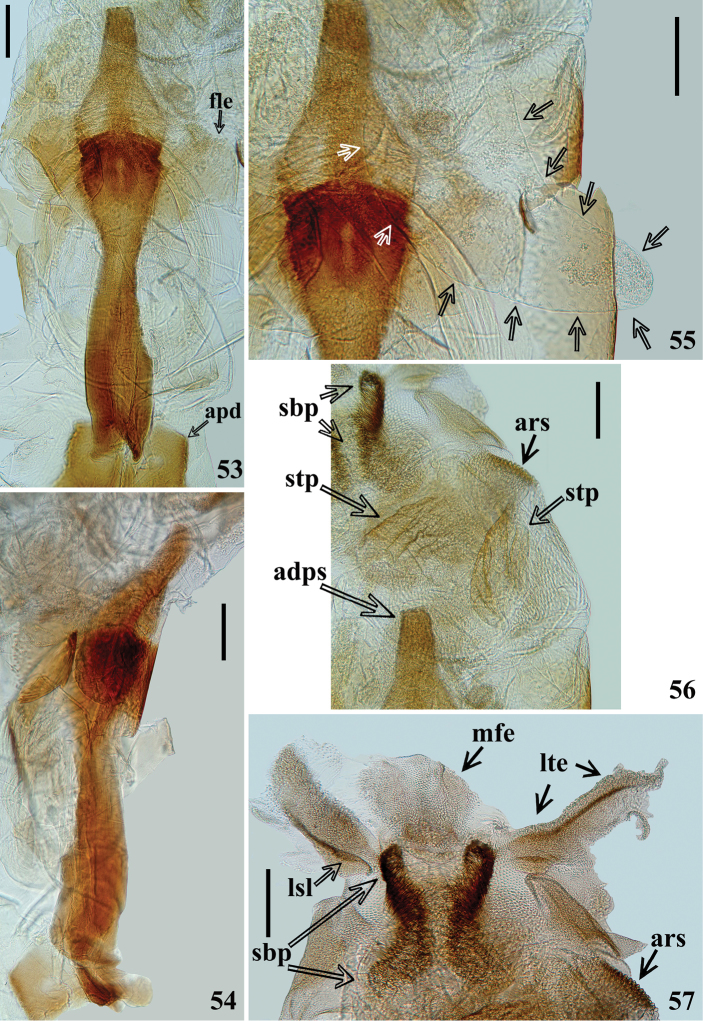
*Volesusnigripennis*, male genitalia **53, 55–57** dorsal view **54** lateral view **53, 54** dorsal phallothecal sclerite (**dps**). (**apd** apex of pedicel; **fle** flat lateral expansion). **55** subapical enlarged portion of dorsal phallothecal sclerite (**dps**) and membranous lobe on endosoma (**mle**), indicated by arrows **56, 57** endosoma portions **56** subapical **57** apical. (**adps** apex of dorsal phallothecal sclerite; **ars** area with rugosities more sclerotized; **mfe** median subrounded flat expansion; **lsl** longitudinal sclerotized line; **lte** lateral tubular short expansion; **sbp** subapical process; **stp** flat, somewhat sclerotized, striated processes). Scale bar: 0.2 mm.

*Male genitalia* (Figs 35, 39–40, 42–57): genital capsule, in ventral and lateral views: exposed portion of pygophore hemispherical, posterior margin (**pm**) flattened, integument rugose and setose; non-exposed portion of pygophore less pigmented and less sclerotized, integument smooth and glabrous (Figs 39, 42–44); in dorsal view: between anterior and posterior genital openings, a very well-sclerotized dorsal (transverse) somewhat curved bridge; socket of insertion of paramere (**sp**) approximately in mid portion of pygophore, with a conspicuous medial prolongation obliquely directed posteriorly (**psp**); numerous, somewhat long, erect setae inserted on inner surface of basal portion of this prolongation; membranous areas of genital opening smooth; proctiger (**pct**) somewhat enlarged toward apex, with numerous long setae on distal half; posterior dorsal margin of pygophore (**pdm**) large, forming a horizontal extension of pygophore wall, with some scattered setae on inner margin and more numerous and somewhat shorter elements on median portion (Fig. 40). Median process of pygophore (**mpp**) only visible in dorsal and lateral views of pygophore, directed upwards, situated some distance from posterior margin, somewhat enlarged, almost straight and subsquared in dorsal and anterior views, respectively (Figs 40, 42–43, 47). Paramere apices in contact in resting position (Fig. 35); parameres (**pa**) (Figs 40, 42–43) symmetrical, elongated, with a lateral rounded enlargement just above inserted portion, moderately and strongly curved inwards at mid and apical portions, respectively, narrowing towards tip, which is somewhat rounded (Fig. 45) to acute (Fig. 46); with straight to moderately curved setae, more numerous towards apical portion; setae absent on basal (inserted) portion and on inner surface of approximately basal fourth of the not inserted portion (Figs 40, 43, 45–46). Articulatory apparatus with moderately short basal plate arms (**bpa**); basal arms and basal plate bridge (**bpb**) forming a subtriangular set (Fig. 50); basal plate bridge (**bpb**) slightly bent ventrally (Fig. 50); pedicel (**pd**) elongated, somewhat enlarged at midportion, curved in lateral view (Figs 48, 49, 51, 52). Before inflation of the endosoma, a lateral oval area (**loa**) somewhat more sclerotized on endosoma wall is evident (Figs 48–50) as well as a conspicuous dorsal pair of membranous lobes on endosoma (**mle**), united at their basal median portion which is inserted just above apex of dorsal phallothecal sclerite (**dps**) (Fig. 50). Each membranous lobe on endosoma (**mle**) is flattened, elongated, apex rounded, directed outwards, laterally to dorsal phallothecal sclerite (Figs 48, 50–52, 55). Dorsal phallothecal sclerite (**dps**) elongated, thrice curved in lateral view (Figs 51, 54); in dorsal view, it is narrower at approximately midportion and towards apical portion (Fig. 53); apical margin almost straight (Figs 50, 52, 53, 56); at its subapical enlarged portion there is a pair of symmetrical rounded flat lateral expansions (**fle**) (Figs 50, 52, 53). After inflation of endosoma, endosoma wall is smooth to longitudinally and transversely finely striated at approximately basal two thirds and coarsely rugose at distal third, with some areas in which the rugosities are more sclerotized (**ars**) (Figs 56, 57); endosoma wall forming three apical expansions: a median subrounded flat expansion (**mfe**) and a pair of lateral tubular short expansions (**lte**), each of the latter with a more sclerotized thin longitudinal line along its length (**lsl**) (Figs 52, 57). Endosoma with the following processes: a pair of flat, somewhat sclerotized, asymmetrical and striated processes (**stp**) between apex of dorsal phallothecal sclerite and subapical process (**sbp**) (Fig. 56). The subapical process (**sbp**) provided with a pair of sclerotized arms, in which basal halves are shorter, diverge more and are formed by stronger sclerotizations of rugosities of wall, while distal half is somewhat longer, less diverging and formed by linear and aggregate thickenings (Figs 51, 52, 56, 57).

###### Distribution.

Colombia, Costa Rica, Ecuador (**new record**), Panama (**new record**).

###### Comments.

*Volesusnigripennis* is the first Sphaeridopinae recorded for Ecuador and Panama (Froeschner 1981, 1999, Maldonado 1990).

The male specimens (Figs 4, 5, 20, 35) described here seem to be generally similar to the female of the species in structure and coloration (Champion 1899, Forero 2006; Figs 1, 2). However, only the examination of more specimens of *V.nigripennis* will make it possible to ascertain whether there is sexual dimorphism.

Smooth areas on the fore lobe of pronotum were recorded here in *V.nigripennis* (Figs 6, 15–17) but it was not possible to distinguish a paired sensory organ similar to that described in *Sphaeridopseulus* by Maldonado and Santiago-Blay (1992: figs 13, 14). These authors emphasized that the nature of the sensory organ of these areas could be seen in their SEM images. However, judging by the SEM images obtained in the present study (Figs 15, 17), it is possible that the supposed sensory organ, also mentioned as present in both species of *Veseris* (Gil-Santana et al. 1999, Gil-Santana and Alencar 2001) may be in fact a portion of these smooth areas. Only future studies, preferably employing histological techniques will allow the evaluation of the existence and/or possible sensory functions of such portions in these species.

Although Champion (1899) had described that the labium would have the second and third visible labial segments equal in length, our studies, including the SEM images, made it clear that the labium is formed by only two visible segments, with the first visible segment short and enlarged and the other long, thin and straight (Figs 11, 12). It is opportune to mention that, according to our request, Dr Dimitri Forero kindly reexamined the female recorded by him from Colombia, sent us photos and confirmed these same features on the labial segments. Similarly, Dr Silvia A. Justi, when examining the female specimen from Panama, also verified that it had only two visible labial segments, with the same characteristics.

Some of the portions of the male genitalia of *V.nigripennis*, such as the parameres and articulatory apparatus, including a basal plate bridge bent ventrally (Figs 40, 45, 46, 50) seem similar to those recorded for species of *Veseris* (Gil-Santana et al. 1999, Gil-Santana and Alencar 2001).

Weirauch (2008) recorded the presence of the basal plate bridge (=ponticulus basilaris) bent ventrad and a pair of membranous lobes on endosoma, lateral to the dorsal phallothecal sclerite in *Sphaeridopsamoenus* and *Salyavatanigrofasciata* Costa Lima, 1935 (Salyavatinae). Judging by her drawings, these lobes are smaller in *S.amoenus* and somewhat larger but shorter in *S.nigrofasciata*, respectively, than those recorded here in *V.nigripennis* (Figs 50–52). It is noteworthy that Weirauch (2008) considered both characteristics (a basal bridge bent ventrad and the pair or membranous lobes on the endosoma) as synamoporphies of the clade Salyavatinae + Sphaeridopinae obtained in her cladistic analysis.

On the other hand, because all other structures, such as those of phallus and endosoma, were not adequately recorded by the above-mentioned authors, nor by others who included just partial or incomplete descriptions of the male genitalia of species of *Sphaeridops* (e.g. Maldonado and Santiago-Blay 1992, Gil-Santana et al. 2000), only future comprehensive studies of these structures among Sphaeridopinae will allow useful comparisons with the results obtained here.

## Discussion

Based on historical evidence and contrary to several authors (e.g. Costa Lima 1940, Wygodzinsky 1949, Maldonado 1990, Forero 2004), we have followed Putshkov and Putshkov (1985) and attributed the authorship of Sphaeridopinae to Amyot and Serville (1843).

The presence of smooth areas on fore lobe of pronotum in between a rugose integument was also recorded in Triatominae, in which its integument “varies from smooth to granular; in many cases, smooth and granular sections occur side by side, forming a characteristic pattern” (Lent and Wygodzinsky 1979). These smooth areas may seem more prominent in Sphaeridopinae, because the surrounding integument is generally much more coarsely rugose.

An unusual characteristic of the group according to Maldonado and Santiago-Blay (1992), the dorsal and ventral components of connexivum well separated by a vertical sclerite, was also recorded to *Volesusnigripennis* (Fig. 34).

However, as commented above, the other alleged unusual characteristic of Sphaeridopinae (Maldonado and Santiago-Blay 1992), i.e., sensory organs on fore lobe of the pronotum, were not seen here in *V.nigripennis*; therefore, the presence of this feature needs more comprehensive studies among species of this group.

On the other hand, although the eyes of Sphaeridopinae have been considered large, almost covering the entire head, nearly contiguous ventrally (Pinto 1927, Maldonado and Santiago-Blay, 1992, Schuh and Slater 1995, Weirauch et al. 2014), this is not the case in *Volesus*. In the latter, the eyes are medium-sized, not covering the head and distant from each other ventrally (Figs 1, 2, 4–14, 20). In fact, the interocular distance is larger than the width of eye, dorsally, and approximately the same of it, ventrally.

Yet, although in the Sphaeridopinae the head had been considered without an anteocular portion (Pinto 1927) or projecting only slightly beyond the anterior margin of eyes (Schuh and Slater 1995), the anteocular portion in *Volesus* is longer, visibly projecting beyond the anterior margin of eyes for almost the same distance as the length of the eye (Figs 8, 11). Lastly, the presence of only two visible labial segments in *Volesus* (Figs 11, 12) is striking.

These dissimilarities between *Volesus* and other genera of Sphaeridopinae suggest that future studies including other species and more specimens, preferably with a phylogenetic approach, should be done in order to ascertain the set of features diagnostic of Sphaeridopinae.

In this case, it is worth mentioning that none of the phylogenetic studies which suggested that Sphaeridopinae would be a sister group to the genus *Salyavata* (Salyavatinae) (Weirauch 2008, Gordon and Weirauch 2016) had included *Volesus* in their analysis.

Therefore, possible future taxonomic changes involving these subfamilies, besides being based on cladistics studies, should also include specimens of *Volesus* to clarify its systematic position within Reduviidae.

In any case, the study of the male of *Volesusnigripennis* allowed for a better definition of the diagnostic characteristics to separate the genera currently considered as valid in Sphaeridopinae. Thus, a revised key to the genera of Sphaeridopinae is presented below.

### Key to the genera of Sphaeridopinae based on Stål (1865, 1872), Gil-Santana and Alencar (2001), Forero (2004) and Gil-Santana et al. (2015)

**Table:** 

1	Length of second visible labial segment equal or subequal to first visible segment; prosternum with a large, rounded to subrounded median excavation	***Veseris* Stål, 1865**
–	Second visible labial segment approximately four to six times longer than first visible segment; prosternum without a rounded to subrounded excavation, but forming a median prolongation or process directed posteriorly for a variable extension between fore coxa	**2**
2	Antenniferous straight apically; labium with only two visible segments; prosternum posteriorly shortly prolonged at midline, not surpassing level of posterior margin of fore coxae and continuous with adjacent sclerite	***Volesus* Champion, 1899**
–	Antenniferous bifurcated apically; labium with three visible segments; prosternum variably prolonged posteriorly, forming a cylindrical median process which surpasses level of posterior margin of fore coxae for a variable extent, distinctly obliquely directed downwards and separated from adjacent sclerite	***Sphaeridops* Amyot & Serville, 1843**

## Supplementary Material

XML Treatment for Volesus

XML Treatment for Volesus nigripennis

## References

[B1] Amyot CJB Serville A (1843) Histoire Naturelle des Insectes. Hémiptères. Librairie Encyclopedique de Roret, Paris, 675 pp.

[B2] Champion GC (1899) InsectaRhynchota. Hemiptera-Heteroptera, Vol II. In: Godman FD Salvin O (Eds) Biologia Centrali Americana.Taylor and Francis, London, 193–304.

[B3] Costa Lima A (1940) Insetos do Brasil. 2 ° Tomo. Capítulo XXII. Hemípteros. Rio de Janeiro, Escola Nacional de Agronomia, 351 pp.

[B4] Dellapé PM Carpintero DL Melo MC (2010) New records of Dipsocoromorpha, Cimicomorpha and Pentatomomorpha (Hemiptera: Heteroptera) from Argentina. Zootaxa 2436: 57–64. 10.11646/zootaxa.2436.1.3

[B5] Dougherty V (1995) A review of the New World Ectrichodiinae genera (Hemiptera: Reduviidae). Transactions of the American Entomological Society 121: 173–225.

[B6] Forero D (2004) Capítulo 5. Diagnosis de los gêneros neotropicales de la família Reduviidae (Hemiptera: Heteroptera), y su distribución em Colombia (excepto Harpactorinae). In: Fernández F Andrade G Amat G (Eds) Insectos de Colombia Vol.3. Universidad Nacional de Colombia, Bogotá DC, 128–275.

[B7] Forero D (2006) New records of Reduviidae (Hemiptera: Heteroptera) from Colombia and other Neotropical countries. Zootaxa 1107: 1–47. 10.11646/zootaxa.1107.1.1 34810786

[B8] Forero D Weirauch C (2012) Comparative genitalic morphology in the New World resin bugs Apiomerini (Hemiptera, Heteroptera, Reduviidae, Harpactorinae). Deutsche Zeitschrift Entomologische 59: 5–41.

[B9] Froeschner RC (1981) Heteroptera or true bugs of Ecuador: a partial catalog. Smithsonian Contributions to Zoology 322: 1–147. 10.5479/si.00810282.322

[B10] Froeschner RC (1999) True bugs (Heteroptera) of Panama: a synoptic catalogue as a contribution to the study of Panamanian biodiversity. Memoirs of the American Entomological Institute, 61: 1–393.

[B11] Gil-Santana HR (2007) New records of Reduviidae (Hemiptera: Heteroptera) from Brazil. Zootaxa 1390: 59–68. 10.11646/zootaxa.1390.1.7

[B12] Gil-Santana HR (2008) New records, and nomenclatural and biological notes on Reduviidae (Hemiptera: Heteroptera) from Bolivia and Brazil. Zootaxa 1785: 43–53.

[B13] Gil-Santana HR Alencar J (2001) Sobre o gênero *Veseris* Stål, 1865, com *Eurylochus* Torre Bueno, 1914, como sinônimo novo e chaves para identificação (Hemiptera, Reduviidae, Sphaeridopinae). Entomología y Vectores 8: 95–104.

[B14] Gil-Santana HR Zeraik SO Costa LAA (1999) Redescrição do macho de *Veserisrugosicollis* (Stål, 1858) (Hemiptera, Reduviidae, Sphaeridopinae). Boletim do Museu Nacional, Nova Série, Zoologia 408: 1–8.

[B15] Gil-Santana HR Costa LAA Zeraik SO (2000) Espécie nova de *Sphaeridops* Amyot & Serville, 1843 (Hemiptera, Reduviidae, Sphaeridopinae). Boletim do Museu Nacional, Nova Série, Zoologia 423: 1–6.

[B16] Gil-Santana HR Forero D Weirauch C (2015) Assassin bugs (Reduviidae excluding Triatominae). In: Panizzi AR Grazia J (Eds) True bugs (Heteroptera) of the Neotropics, Entomology in Focus 2.Springer Science+Business Media, Dordrecht, 307–351. 10.1007/978-94-017-9861-7_12

[B17] Gordon ERL Weirauch C (2016) Efficient capture of natural history data reveals prey conservatism of cryptic termite predators. Molecular Phylogenetics and Evolution 94: 65–73. 10.1016/j.ympev.2015.08.015 26314664

[B18] Inés OV Coscarón MC (2009) Additional records of Heteroptera (Hemiptera) from Argentina. Zootaxa 2311: 38–48.

[B19] Lent H Wygodzinsky P (1979) Revision of the Triatominae (Hemiptera: Reduviidae) and their significance as vectors of Chagas’ disease. Bulletin of the American Museum of Natural History 163: 123–520.

[B20] Lethierry L Severin G (1896) Catalogue général des Hémiptères. Tome III. Hétéroptères. R. Friedländer & Fils, Libraires-Éditeurs, Berlin, 275 pp.

[B21] Maldonado CJ (1990) Systematic Catalogue of the Reduviidae of the World. Caribbean Journal of Science, Special publication No. 1, University of Puerto Rico, Mayagüez, 694 pp.

[B22] Maldonado CJ Santiago-Blay JA (1992) A new species of the Neotropical genus *Sphaeridops* Amyot & Serville, 1843 (Sphaeridopinae: Reduviidae). Proceedings of the Entomological Society of Washington 94: 508–511.

[B23] Martin-Park A Delfín-González H Coscarón MC (2012) Revision of genus *Repipta* Stål 1859 (Hemiptera: Heteroptera: Reduviidae: Harpactorinae) with new species and distribution data. Zootaxa 3501: 1–54.

[B24] Melo MC (2008) New records of Peruvian Reduviidae (Heteroptera), with the description of a new species of *Tagalis* Stål 1860 (Saicinae). Zootaxa 1763: 55–62.

[B25] Pinto C (1927) Sphaeridopidae, nova familia de Hemiptera Reduvioideae, com a descripção de um genero e especie nova. Boletim Biologico 6: 43–51.

[B26] Putshkov VG Putshkov PV (1985) A catalogue of the assassin-bugs genera of the world (Heteroptera, Reduviidae). Published by the authors, Kiev, 137 pp.

[B27] Rédei D Tsai J-F (2011) The assassin bug subfamilies Centrocnemidinae and Holoptilinae in Taiwan (Hemiptera: Heteroptera: Reduviidae). Acta Entomologica Museu Nationalis Pragae 51: 411–442.

[B28] Rosa JA Mendonça VJ Rocha CS Gardim S Cilense M (2010) Characterization of the external female genitalia of six species of Triatominae (Hemiptera, Reduviidade) by scanning electron microscopy. Memórias do Instituto Oswaldo Cruz 105: 286–292. 10.1590/S0074-02762010000300007 20512241

[B29] Rosa JA Mendonça VJ Gardim S Carvalho DB Oliveira J Nascimento JD Pinotti H Pinto MC Cilense M Galvão C Barata JMS (2014) Study of the external female genitalia of 14 *Rhodnius* species (Hemiptera, Reduviidae, Triatominae) using scanning electron microscopy. Parasites & Vectors 7: 17. 10.1186/1756-3305-7-17 PMC3896706 24405517

[B30] Schuh RT Slater JA (1995) True bugs of the world (Hemiptera: Heteroptera): classification and natural history. Cornell University Press, Ithaca, NY, 336 pp.

[B31] Stål C (1865) Hemiptera africana. Tomo III. Officina Norstedtiana, Holmiae, 275 pp.

[B32] Stål C (1872) Enumeratio Reduviinorum Americae. In: Enumeratio Hemipterorum. Kongliga Svenska Vetenskaps-Akademiens Handlingar 10: 66–128.

[B33] Walker F (1873a) Catalogue of the specimens of HemipteraHeteroptera in the collection of the British Museum (Part VII). Printed for the Trustees of the British Museum, London, 213 pp.

[B34] Walker F (1873b) Catalogue of the specimens of HemipteraHeteroptera in the collection of the British Museum (Part VIII). Printed for the Trustees of the British Museum, London, 220 pp.

[B35] Weirauch C (2008) Cladistic analysis of Reduviidae (Heteroptera: Cimicomorpha) based on morphological characters. Systematic Entomology 33: 229–274. 10.1111/j.1365-3113.2007.00417.x

[B36] Weirauch C Bérenger J-M Berniker L Forero D Forthman M Frankenberg S Freedman A Gordon E Hoey-Chamberlain R Hwang WS Michael A Udah O Watson C Zhang G Zhang J (2014) An illustrated identification key to assassin bug subfamilies and tribes (except Emesinae). Canadian Journal of Arthropod Identification 26: 1–115. 10.3752/cjai.2014.26

[B37] Wygodzinsky P (1949) Elenco sistematico de los reduviiformes americanos. Instituto de Medicina Regional de la Universidad Nacional de Tucumán, Monografia 1: 1–102.

[B38] Zhang G Hart ER Weirauch C (2016) A taxonomic monograph of the assassin bug genus *Zelus* Fabricius (Hemiptera: Reduviidae): 71 species based on 10,000 specimens. Biodiversity Data Journal 4: e8150. 10.3897/BDJ.4.e8150 PMC5019016 27651730

